# Self-Mixing Demodulation for Coherent Phase-Sensitive OTDR System

**DOI:** 10.3390/s16050681

**Published:** 2016-05-12

**Authors:** Haijun He, Li-Yang Shao, Zonglei Li, Zhiyong Zhang, Xihua Zou, Bin Luo, Wei Pan, Lianshan Yan

**Affiliations:** Center for Information Photonics & Communications, School of Information Science and Technology, Southwest Jiaotong University, Chengdu 610031, China; haijhe@163.com (H.H.); liz.onglei@163.com (Z.L.); zhiyongzhang@home.swjtu.edu.cn (Z.Z.); zouxihua@swjtu.edu.cn (X.Z.); bluo@home.swjtu.edu.cn (B.L.); wpan@home.swjtu.edu.cn (W.P.); lsyan@home.swjtu.edu.cn (L.Y.)

**Keywords:** fiber optics sensors, remote sensing and sensors, scattering measurement, optical time domain reflectometry

## Abstract

Phase-sensitive optical time domain reflectometry (Ф-OTDR) attracts much attention due to its capability of telling the type and position of an intrusion simultaneously. In recent decades, coherent Ф-OTDR has been demonstrated to realize long-distance detection. For coherent Ф-OTDR, there are three typical demodulation schemes in the reported studies. However, they still cannot realize real-time monitoring to satisfy practical demands. A simple and effective demodulation method based on self-mixing has been put forward to demodulate the beat signal in coherent Ф-OTDR. It not only saves a local electrical oscillator and frequency locked loop, but also demodulates the beat signal without residual frequency. Several vibrations with different frequency were separately applied at the same location of a 42.5 km fiber. The spatial resolution of 10 m and frequency response range from 8 Hz to 980 Hz have been achieved. The precise location with signal-to-noise ratio of 21.4 dB and broadband measurement demonstrate the self-mixing scheme can demodulate the coherent Ф-OTDR signal effectively.

## 1. Introduction

Conventional optical time domain reflectometry (OTDR) has been widely used in optical fiber communication to detect fault events of fiber links. The working principle is to measure the index of fraction changes [1,2,3]. However, only static changes can be discerned, such as the fiber attenuation, splice and connector loss and break points. Dynamic events cannot be determined by using conventional OTDR. Moreover, its spatial resolution is generally tens of meters, which is not enough to locate the position of a fault precisely. With the capability of telling the type and exact position of an intrusion simultaneously, phase-sensitive optical time domain reflectometry (Ф-OTDR) has attracted much attention since its invention [4]. Furthermore, Ф-OTDR has many field applications such as in the health monitoring of large-scale structures, including borders, bridges, tunnels, oil pipes and so on [5,6].

There are two typical signal detection schemes in the Ф-OTDR system including direct detection [7,8,9,10] and coherent detection [11,12,13,14,15,16,17,18,19,20]. In the direct detection scheme, the setup is simple; only the intensity of the optical signal is detected by using a high-sensitivity photodetector. However, the sensing distance is limited (without amplification). To achieve a longer sensing distance, a complex structure and high cost (extra pump laser) are necessary for the direct detection scheme [9,10]. Different from the direct detection technique, local light and a balanced photodetector are used to implement the longer sensing distance in the coherent detection scheme. This greatly reduces the complexity and the cost of the whole system. Up until now, there were three kinds of signal demodulation methods for the system based on coherent detection, including the zero-span function of the electrical spectrum analyzer (ESA) [11,12], heterodyne detection [13,14,15,16,17,18] and digital coherent detection [19,20]. In previous reports, the zero-span function of the ESA was used to eliminate all the other unwanted electrical components to obtain a pure signal [11,12]. However, it is not practical in real-time monitoring and the ESA increases the cost of the system greatly. In the heterodyne demodulation scheme, a stably local electrical signal mixes with the beat signal where its frequency strictly equals the frequency shift caused by the acoustic-optic modulator (AOM) [13,14,15,16,17,18]. However, this synchronous demodulation scheme is sensitive to the variation of the beat signal, and typically a frequency locked loop is needed. The instability of the beat signal makes the signal-to-noise ratio (SNR) of the detection system fluctuate because of the frequency drift of the laser and the AOM [16,17]. For the demodulation method of digital coherent detection, a data acquisition card (DAQ) with an ultra-high sample rate and a large amount of storage is necessary. That makes too much unnecessary data be stored and processed. Although it can get a better performance in demodulation, it takes much more time and cannot realize real-time monitoring [19,20].

In this work, a self-mixing signal demodulation scheme has been proposed for the coherent Ф-OTDR system. It extracts the intrusion signal from the beat signal by mixing the beat signal itself, which avoids generating a local signal and a frequency locked loop compared to the heterodyne detection. More importantly, it eliminates the residual frequency (caused by the instability of the beat signal) to achieve a pure signal. Thus, it greatly improves the demodulation performance. The experimental results prove the feasibility of this technique by simultaneously determining the frequency and location of the vibration intrusion. A 10 m spatial resolution has been achieved on a 42.5 km fiber link. In addition, the detectable frequency range from 8 Hz to 980 Hz and a high SNR of 21.4 dB have been implemented in this work.

## 2. Principle

**Figure 1:**
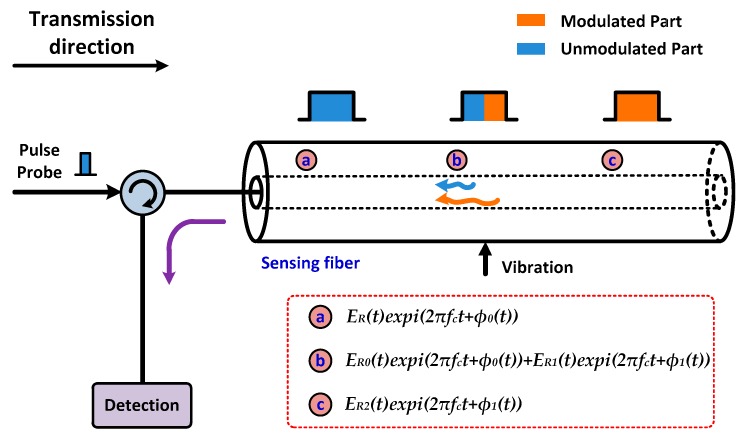
The theory description of the phase-sensitive OTDR.

The phase of the backscattered Rayleigh light carries the vibration information when an optical pulse from a high-coherence laser source is injected into a sensing fiber [7,8,17,21]. The optical signals will interfere with each other within one pulse duration. They will interfere when the backscattered signal of the pulse leading edge (embracing the vibration information) meets with the signal behind it which has no vibration information [8,17,21]. The evolution of the backscattered Rayleigh light within a one-pulse duration is shown in Figure 1, including the unmodulated, partly modulated and completely modulated signal [8,17,21]; the part of the signal with the vibration information is called modulated (orange part) while that without vibration information is called unmodulated (blue part). It means that the phase difference of two backscattered signals within a one-pulse duration reflects the external information (vibration/strain). The backscattered Rayleigh light returns to the end of the pulse light injection, which can be described as:

(1) $$E(t)=E_{R1}(t)\cdot \exp[i(2\pi f_{c}t+\phi_{1}(t))]+E_{R0}(t)\cdot \exp[i(2\pi f_{c}t+\phi_{0}(t))]$$

The phases of two backscattered optical signals are *φ*_1_(*t*) which carries vibration information and *φ*_0_(*t*) which is the normal backscattered one (without vibration information). *E_R_*_1_(*t*) and *E_R_*_0_(*t*) are the amplitudes, correspondingly, and *f_c_* is the center frequency of the light output from the laser source.

For the direct detection method, these two parts will interfere at the photodetector. The output of the photodetector *I_direct_*(*t*) is expressed by [21].

(2) $$I_{direct}(t)\;\propto \;E_{R1}^{2}(t)+E_{R0}^{2}(t)+2\cdot E_{R1}(t)\cdot E_{R0}(t)\cdot \cos(\phi_{1}(t)-\phi_{0}(t))$$

**Figure 2:**
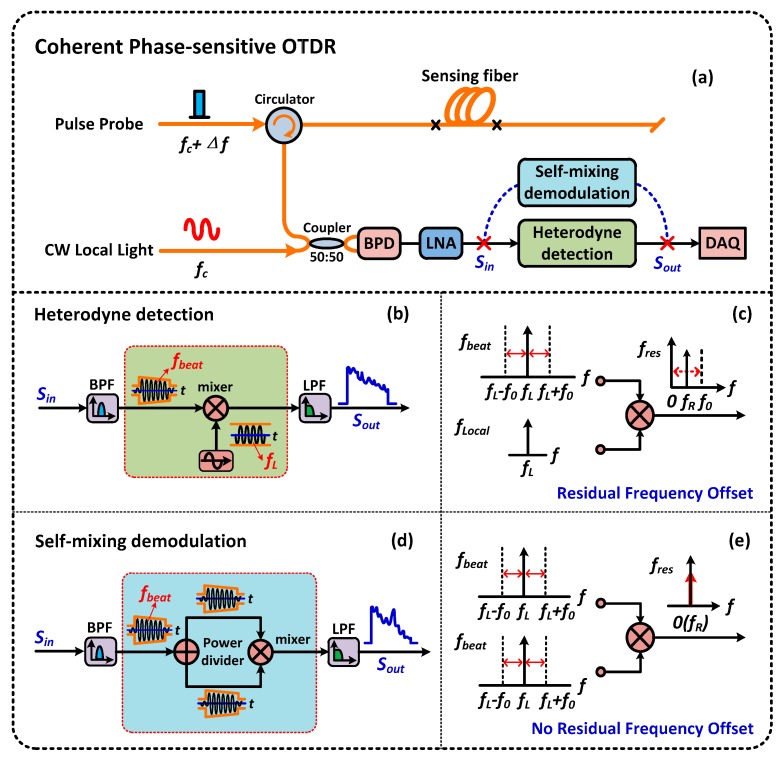
The principle comparison between the self-mixing and the heterodyne demodulation method. (**a**) The schematic diagram of the coherent phase-sensitive OTDR system based on self-mixing demodulation and heterodyne detection; BPD: Balanced photo detector; LNA: Low noise amplifier; BPF: Bandpass filter; LPF: Low-pass filter; DAQ: Data acquisition card; (**b**) The schematic diagram and (**c**) the demodulated result of the heterodyne detection; (**d**) The schematic diagram and (**e**) the demodulated result of the self-mixing demodulation.

To realize long-distance sensing with a satisfying SNR, the Ф-OTDR system based on coherent detection has been proposed [11,12,13,14,15,16,17,18,19,20]. As shown in Figure 2a, the pulse probe light modulated by an AOM is injected into a sensing fiber to detect the external vibration/intrusion. The backscattered Rayleigh light can be described as *E_R_*_1_(*t*)*expi*(*2π*(*f_c_* + *∆f*)*t* + *φ*_1_(*t*)) + *E_R_*_0_(*t*)*expi*(*2π*(*f_c_* + *∆f*)*t* + *φ*_0_(*t*)), and *∆f* is the frequency shift introduced by the AOM. The backscattered Rayleigh light combines with a continuous wave (CW) local light by a 3 dB coupler before it falls on the balanced photodetector. The CW local light *E_LO_*(*t*)*expi*(*2πf_c_t*) is split from the coherent laser through a 90:10 coupler. The coupled light is detected by a balanced photodetector. The detected current *I_coherent_* is proportional to the optical power.

(3) $$\begin{array}{l} I_{coherent}(t)\;\propto \;E_{R1}^{2}(t)+E_{R0}^{2}(t)+E_{LO}^{2}(t)+2\cdot E_{R1}(t)\cdot E_{R0}(t)\cdot \cos(\phi_{1}(t)-\phi_{0}(t))\\ \;+2\cdot E_{LO}(t)\cdot \cos\theta (t)\cdot [E_{R1}(t)\cdot \cos(2\pi \Delta ft+\phi_{1}(t))+E_{R0}(t)\cdot \cos(2\pi \Delta ft+\phi_{0}(t))] \end{array}$$

where *E_LO_*(*t*) is much larger than the *E_R_*_1_(*t*) and *E_R_*_2_(*t*), and *θ*(*t*) is the relative polarization angle between the backscattered light and the CW local light.

In previous reports [13,14,15,16,17,18], the schematic diagram of coherent Ф-OTDR based on heterodyne detection is illustrated in Figure 2b. The AC component of *I_coherent_*(*t*) in Equation (3) can be obtained by passing through a carefully selected electrical bandpass filter or high-pass filter, which can be rewritten as:

(4) $$S_{AC}(t)\;\propto \;2\cdot E_{LO}(t)\cdot \cos\theta (t)\cdot [E_{R1}(t)\cdot \cos(2\pi \Delta ft+\phi_{1}(t))+E_{R0}(t)\cdot \cos(2\pi \Delta ft+\phi_{0}(t))]$$

In the heterodyne detection scheme, a local electrical oscillator with extremely stable frequency *∆f* is necessary to obtain the demodulated signal of *2E_LO_*(*t*)*cosθ*(*t*)[*E_R_*_1_(*t*)*cosφ*_1_(*t*) + *E_R_*_0_(*t*)*cosφ*_0_(*t*)]. Meanwhile, the frequency *∆f* must be equal to the center frequency of the beat signal. Unfortunately, the center frequency is unstable because of the frequency drift of the local light and the jitter of the AOM driver [13,14,15,16,17,18]. Although the local electrical oscillator is stable enough, the demodulated signal cannot eliminate the frequency component completely, as illustrated in Figure 2c. Thus, a typical frequency locked loop is needed to follow the beat signal variation tightly [13,14,15,16,17,18]. In addition, the demodulated signal does not contain a phase difference since the two components in Equation (4) do not interfere with each other. That makes further data processing necessary (to realize the precise location and frequency measurement).

Compared to the heterodyne detection method, the self-mixing demodulation technique extracts the intrusion signal from the beat signal by mixing the beat signal itself, as shown in Figure 2d. It avoids generating a local electrical signal and setting up a frequency locked loop in addition. More importantly, although the frequency of the beat signal is unstable, it can demodulate the beat signal completely (without residual frequency) and obtain the phase difference. Figure 2e shows the demodulated result in the situation of the beat signal instability. Obviously, there is no residual frequency left since the mixing signals are split from the same signal. Therefore, this method of self-mixing can overcome the defect in heterodyne detection. The demodulation result of *S_self-mixing_*(*t*) can be described as:

(5) $$S_{self-mixing}(t)\;=\;S_{AC}(t)\times S_{AC}(t)$$

The vibration information can be extracted by using a deliberately selected low-pass filter with suitable cutoff frequency.

(6) $$S_{self_{-}LPF}(t)\;=\;2\cdot {\left[\cos\theta (t)\cdot E_{LO}(t)\right]}^{2}\cdot [E_{R1}^{2}(t)+E_{R0}^{2}(t)+2\cdot E_{R1}(t)\cdot E_{R0}(t)\cdot \cos(\phi_{1}(t)-\phi_{0}(t))]$$

For a stable laser, the *E_LO_*(*t*) is almost a constant, and the change of the relative polarization angle *θ*(*t*) is a slow change process compared with the dynamic measurement [12,13]. Therefore, the coefficient 2[*cosθ*(*t*)*E_LO_*(*t*)]^2^ is a slowly varying variable. It can be considered as a constant in comparison with the vibration signal. Compared to *I_direct_*(*t*) in Equation (2), only the coefficients are different. So the self-mixing scheme can extract the vibration information modulated on the optical signal in theory.

## 3. Experimental Setup

**Figure 3:**
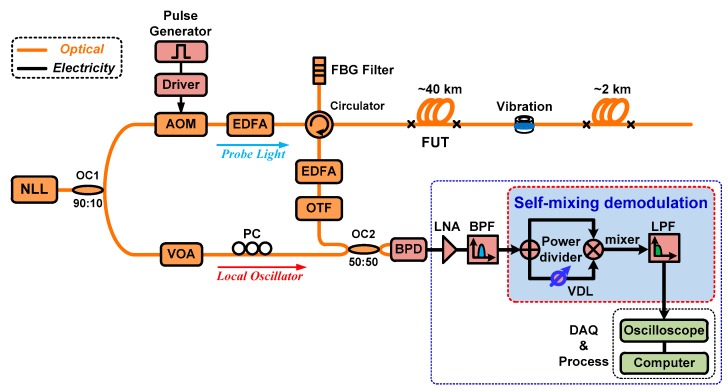
Experimental setup of coherent phase-sensitive OTDR system based on the self-mixing demodulation scheme. NLL: Narrow linewidth laser; OC: Optical coupler; AOM: Acoustic-optic modulator; EDFA: Erbium-doped fiber amplifier; FBG: Fiber Bragg grating; FUT: Fiber under test; OTF: Optical tunable filter; VOA: Variable optical attenuator; PC: Polarization controller; BPD: Balanced photo detector; LNA: Low noise amplifier; BPF: Bandpass filter; VDL: Variable delay line; LPF: Low-pass filter.

Figure 3 shows the setup of the coherent Ф-OTDR system based on the self-mixing demodulation technique. The linewidth of the laser source is ~3 kHz. The center wavelength of the laser is 1550.09 nm and the optical output peak power is 20 mw. The narrow linewidth laser is split into two branches through a 90:10 fiber coupler. The upper path is modulated into an optical pulse by an AOM with a frequency shift of 200 MHz, acting as a probe light. The repetition rate of the modulated light pulse is 2 kHz and the pulse width is 100 ns. After amplifying and filtering by a pulse-Erbium doped fiber amplifier (EDFA) and fiber Bragg grating (FBG) filter consecutively, the light pulse is injected into 42.5 km test fiber through an optical circulator. The backscattered Rayleigh signal is amplified and filtered by an EDFA and optical tunable filter (OTF), respectively. The CW reference light in the lower path is used as the local oscillator which is combined with the backscattered Rayleigh light after adjusting its intensity and polarization angle by a variable optical attenuator (VOA) and polarization controller (PC), respectively. The alternating current (AC) component of the beat signal is detected by a balanced photodetector. After amplifying and filtering by a low noise amplifier (LNA) and bandpass filter (BPF) consecutively, the electrical signal is divided into two parts through a 3 dB power divider. Then, the two signals interact with each other by being injected into a mixer after adjusting the time delay by a variable delay line (VDL). The mixing signal is filtered by a low-pass filter (LPF) with a cut-off frequency of 20 MHz. Lastly, the demodulated signal is sampled by an oscilloscope with a sample rate of 100 MS/s and processed with a personal computer.

## 4. Experimental Results

**Figure 4:**
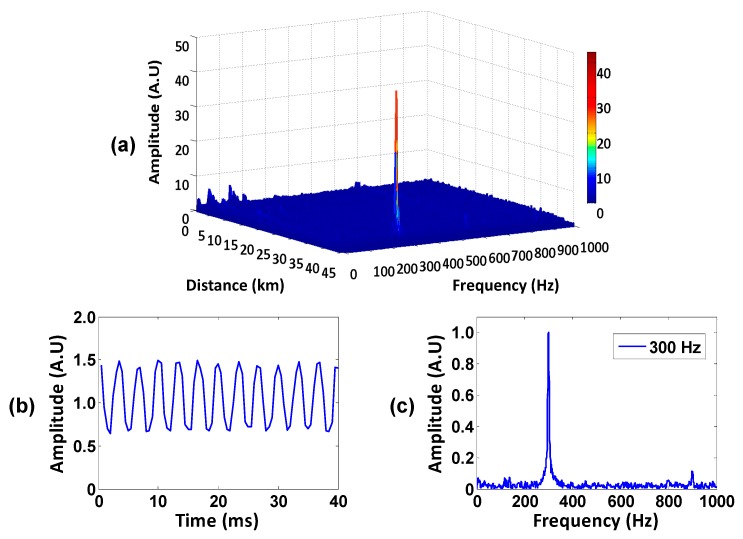
(**a**) The auto-power spectrum of vibration along the sensing fiber; (**b**) the time domain signal and (**c**) the frequency spectrum of the vibration with the peak of 300 Hz.

A vibration with a frequency of 300 Hz was applied to the sensing fiber. Three hundred consecutive traces were sampled by an oscilloscope (with a sample rate of 100 MS/s). By calculating the auto-power spectrum of vibration along the sensing fiber, the vibration with a frequency of 300 Hz appears at 40.45 km clearly, as illustrated in Figure 4a. Figure 4b,c show the time domain signal and the corresponding frequency spectrum at the test point, respectively. Limited by the performance of the vibration actuator (PZT) and non-treatment of the signal, the time domain curve is slightly fluctuant. Note that all results are not processed with the filtering algorithm. Obviously, the demodulation results are in good agreement with the applied vibration signals.

**Figure 5:**
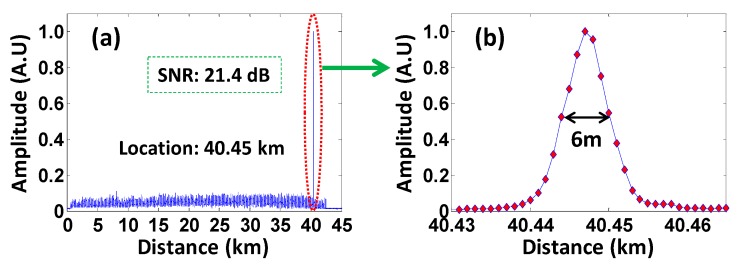
(**a**) The location and SNR of the vibration; (**b**) the achieved spatial resolution of this system.

By subtracting the amplitude traces from the first trace and computing the sum of the absolute amplitude change [11,12], the vibration location curve is shown in Figure 5a. The achieved SNR of the intrusion signals reaches up to 21.4 dB at the vibration position of 40.45 km (the SNR is calculated as the ratio between the peak intensity of the vibration signal and the root-mean-square intensity of the background noise) [11]. The vibration was applied along 1.5 m of fiber at the vibration position. In the experimental results, one point represents one meter since the sample rate of 100 MS/s was used in these experiments. Figure 5b shows that the full width of the vibration location is about 12 m (40,446–40,458 m). That means the spatial resolution is ~12 m without any further processing [22]. Considering the pulse-broadening caused by the AOM (the optical pulse modulated by the AOM has a rising edge over 10 ns), it is in good agreement with the probe pulse width of 100 ns [22].

**Figure 6:**
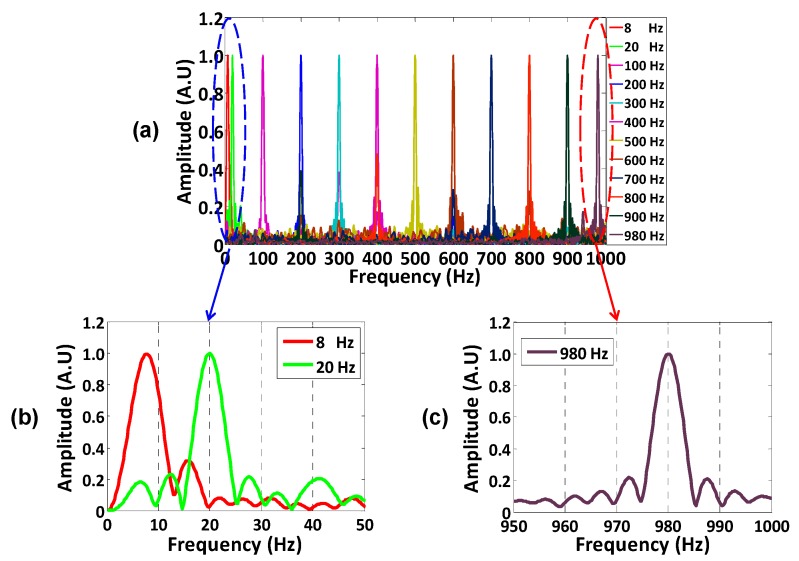
The frequency spectrum measured by different vibration signals. (**a**) The frequency spectrums tested in experiment; (**b**) the applied minimum frequency and (**c**) the applied maximum frequency.

Restricted by the fiber length of 42.5 km, the pulse repetition of 2 kHz was used in the experiment. Therefore, the maximum frequency response is 1 kHz according to the Nyquist theorem. To identify the capability of broadband measurement, the vibration signals with a frequency from 8 Hz to 980 Hz were applied on the sensing fiber separately. Figure 6a shows the normalized power spectrum with a frequency range from 8 Hz to 980 Hz. Figure 6b,c show the details of the applied minimum and maximum frequency, respectively.

## 5. Discussions

### 5.1. The Limitation of the Sensing Distance Using the Self-Mixing Method

**Figure 7:**
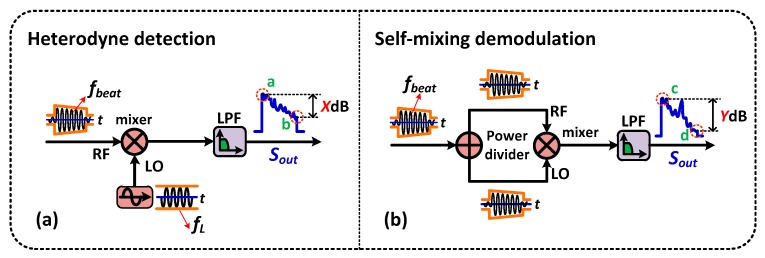
The comparison between the heterodyne detection and the self-mixing method on the maximum sensing distance. (**a**) The heterodyne detection and (**b**) the self-mixing demodulation.

Although the self-mixing method can demodulate the beat signal effectively, its maximum sensing distance is shorter than that of the heterodyne detection method (for the same experimental setup and parameters). To achieve the same maximum sensing distance, the gain of the LNA or the optical power in self-mixing should be larger than the heterodyne detection. Besides, the mixer works effectively when the power of the local oscillator lies in the working range. Therefore, a high-gain LNA is needed to amplify the local oscillator. In addition, the maximum sensing distance is also limited by the width of the mixer’s dynamic range. As shown in Figure 7a, a stable local oscillator mixes with the beat signal, and the output of the mixer is proportional to the Radio Frequency (RF) signal (*f_beat_*). We assume that the difference between the maximum and minimum value is *X* dB, which is the dynamic range of the mixer. For the self-mixing method, the difference value changes into *Y* dB, as illustrated in Figure 7b. It is equal to *2X* dB. Thus, the self-mixing method will reduce the maximum sensing distance to half (for the same mixer). To achieve a longer maximum sensing distance, a mixer with a large dynamic range and high-gain LNA are necessary.

### 5.2. Robustness of Self-Mixing Demodulation

**Figure 8:**
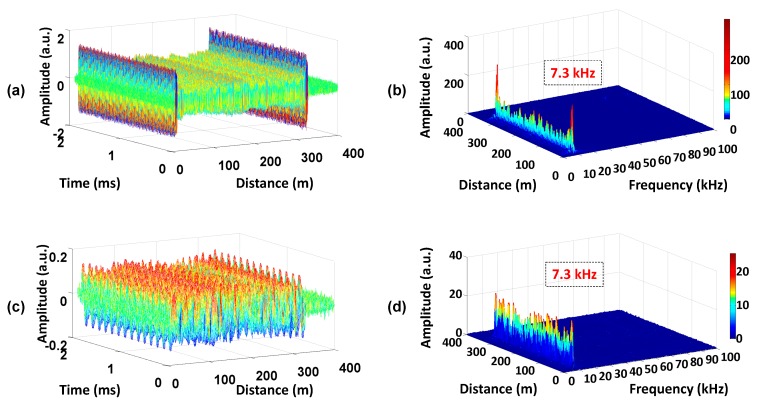
The variation frequency of the beat signal. (**a**) The traces of the beat signal at different periods and (**b**) the power spectrum along the sensing fiber; (**c**) The traces at different periods and (**d**) the power spectrum along the sensing fiber after mixing a cosine signal with the fixed frequency.

Another important issue is how fast the self-mixing method can deal with the frequency variation of the optical signal itself. In this system, the frequency shift of the laser source has negligible effect on the beat signal because the change in the laser source is much slower than the optical pulse. So the frequency variation of the beat signal is mainly caused by the frequency drift of the AOM [16,17]. Additionally, it cannot be adjusted, but the variation value may be measured. A simple setup has been adopted to obtain the beat signal and calibrate the frequency shift of the AOM [23]. The fiber length is set to be 320 m, which determines the return time and period of the optical pulse. The maximum detectable variation frequency is 100 kHz with the pulse period of 5 µs. The results are shown in Figure 8 and they were tested without interference (to obtain the correct variation frequency). The three-dimensional figure of Time-Distance presents the beat signal (with the center frequency of 200 MHz) sampled at different periods, illustrated in Figure 8a. Figure 8b is calculated with the time domain signal in Figure 8a at the same position. For example, the frequency peak at 100 m (in Figure 8b) is calculated with the 2 ms time domain signal at 100 m (in Figure 8a). Similarly, Figure 8c,d are calculated with the baseband signal (mixing with a cosine signal with a fixed frequency of 200 MHz). Obviously, two intense peaks with same frequency of 7.3 kHz along the whole link are shown in Figure 8b,d under the condition of no interference, respectively. The same variation frequency measured by the two signals (with different center frequency) proves the reliability of the measured value. Therefore, the variation frequency of the beat signal is ~7.3 kHz. As shown in Figure 4, there is no other intense peak except for the applied frequency peak. That indicates the self-mixing method can overcome the frequency variation of 7.3 kHz at least.

## 6. Conclusions

In this paper, we propose a self-mixing method to extract the vibration signal modulated in the Rayleigh backscattered signal in the coherent Ф-OTDR system. Compared to previous methods, the self-mixing demodulation technique extracts the intrusion signal from the beat signal by mixing the beat signal itself, which avoids generating a local signal and a typical frequency locked loop as well. More importantly, this method can eliminate the residual frequency caused by the frequency drift of the local oscillator and the AOM driver. Thus, a superior demodulated signal can be achieved with this method.

In this work, we have experimentally demonstrated the demodulation capability of the self-mixing method. With 100 ns pulse-width modulated light, the spatial resolution of 10 m has been achieved. The vibration location on a 42.5 km fiber has been precisely obtained with a satisfying SNR of 21.4 dB. Meanwhile, more than 10 vibration signals with different frequency from 8 Hz to 980 Hz were measured accurately.
